# Progress in Gynæcology

**Published:** 1900-05-12

**Authors:** 


### PROGRESS IN GYNÆCOLOGY.

**The Treatment of Sterility.**—Frederick Edge,[1] after discussing the various causes of sterility, says that while in some cases the cause is very obvious and the treatment simple, in many others it is very obscure, and unless some definite method be employed it is not to be expected that all, or rather the necessary amount of, hindrance will be removed to enable conception to take place. When nothing definite can be made out by ordinary examination, he recommends the following procedure: The patient is anæsthetised and placed on the operation table in the lithotomy position; the vulva and vagina thoroughly scrubbed and cleaned with 1-100 lysol solution, methylated spirit, and 1-1,000 corrosive sublimate solution. The vulva is now carefully examined, and such impediments to coitus as caruncles, lipomata, &c., are removed. Remnants of the hymen causing vaginismus are dealt with, the whole hymeneal insertion being taken away. Abnormal length of labia and clitoris may be treated by a plastic operation. The vaginal introitus, if found tight and narrow, is forcibly dilated with the fingers. The vagina is now examined, and any cysts or tumours in its wall attacked. If the perineum is very torn or deficient it must be repaired. The cervix and os uteri are examined, and any uterine displacement corrected. If the cervix be elongated, it is necessary to amputate it, and if follicular hypertrophy be present keel-shaped excision may be done. If the patient is a nullipara the os may be stenosed, but whether stenosed or not, the method advised is to dilate the cervix, to amputate if necessary, and to perform posterior discision. The dilatation of the cervix enables any tumour of the uterus or diseased states of the endometrium to be made out and appropriate treatment adopted. Having dilated and divided the cervix, and curetted the endometrium, the next step in these cases of indefinite and obscure causation of sterility is to deal with the Fallopian tubes and ovaries. For this purpose Edge advises anterior vaginal cœliotomy, so that the fundus of the uterus, with the tubes and ovaries, may be drawn into view. The tubes are carefully examined, constructing bands divided, kinks straightened out, and agglutinated fimbræ opened up. The ovaries are freed from adhesions and cysts, or portions of diseased ovarian tissue excised. Lefèvre,[2] writing on the same subject, lays stress on the fact that sterility often depends on several pathological conditions being present, and unless treatment is directed to all these complications failure will be certain.

**Backward Displacements of the Uterus.**—Thomas Wilson[3] recognises three varieties of backward displacement of the uterus: (1) Retroposition, in which the organ is simply moved backwards without any change of inclination; (2) retroversion, in which the organ is rotated backwards round a transverse axis drawn through the internal os; (3) retroflexion or bending of the womb, so that its axis is concave backwards. He maintains that flexion depends upon changes in the organ itself, while alteration in position depends upon affections of the neighbouring and supporting tissues; he thinks that in the common backward displacements with which we have to deal retroversion is usually the first change, and that bending does not take place until after some time has elapsed. He also holds the view that mobile retroversions of the uterus are among the commonest affections peculiar to women. In discussing the natural relations of the uterus, he says they cannot be properly studied in the post-mortem room, because most commonly a marked alteration in position occurs after death. The uterus, with its axis a little curved forwards, normally lies midway between the plane of the pelvic inlet and that of the outlet, and under normal conditions allows of a considerable degree of passive mobility. As the bladder gradually fills, the womb is carried upwards and backwards, and its body at the same time turns backwards until the axis of the organ is nearly or quite vertical; when the rectum is distended the uterus is elevated and partly retroverted. In every case of backward displacement the uterus is supported at a lower level than the normal one, and this lowered position is allowed by yielding or relaxation of the supports of the uterus. The pelvic floor is the most important agent in supporting the uterus at its proper level. While the uterus is thus maintained at its normal level in a position of anteversion, free mobility of the organ is allowed. The portion of the uterus which is least movable is the supravaginal cervix, round which there is a considerable collection of connective tissue. From the cervix three pairs of notable bundles of connective tissue run, one pair anteriorly round the base of the bladder on either side, another pair backwards and upwards on either side of the rectum, and the third directly outwards at the bases of the broad ligaments. In the healthy adult female the pelvic connective tissue contains a considerable quantity of yellow elastic fibres, and much involuntary muscular tissue; the pelvic floor also consists essentially of muscular and connective tissue. The muscular tissue is actively contractile, and by its tone is peculiarly adapted for supporting pressure; the connective tissue and fasciæ act by passive elasticity, and are prone after long continued pressure or stretching to become elongated permanently. Wilson considers that the alterations caused in the supporting tissues by labour and abortion are by far the most important agents in allowing retroversion of the uterus to take place.

Oliver,[4] in a paper on version of the uterus, points out that the peritoneum is a highly elastic structure, and that that portion of it which envelops the uterus, and enters into the formation of the broad ligaments, is in its natural connection passively extended, and that this passive extension plays an important part in maintaining the natural inclination of the uterus. When the elasticity of the serous covering is unduly diminished or lost, the organ is less capable of withstanding the influence of adverse forces, and assumes that position which the resultant of these might determine. Wilson considers the treatment of these cases under two heads—preventive and remedial. Preventive depends on the correct management of labour and the lying-in period, because everything that promotes involution of the uterus favours the proper involution of its supports, and tends to prevent backward displacement. Remedial treatment consists of first reducing the uterus to its proper position, and, secondly, maintaining it in that position. He considers that reduction is best effected by the hands, and when this is accomplished it is best kept up by some sort of pessary—Hodge's sigmoid, or Schultze's figure of 8. When operative measures are called for there is a choice of three different methods—(1) Alexander's operation of shortening the round ligaments; (2) abdominal fixation; (3) anterior fixation of the uterus performed through the vagina. Of these he prefers for mobile retroversion that of anterior vaginal fixation.

[1] Birmingham Med. Rev., Nov., 1899. [2] Brit. Med. Jour., Oct., 1899. [3] Birmingham Med. Rev., Jan., 1900. [4] Brit. Med. Jour., Nov. 18, 1899.

